# Secondary hemophagocytic lymphohistiocytosis associated with heat stroke: A case report and review of literature

**DOI:** 10.1097/MD.0000000000033842

**Published:** 2023-05-26

**Authors:** Wongook Wi, Kyoung Won Yoon, Hyo Jin Kim

**Affiliations:** a Department of Anesthesiology and Pain Medicine, Chung-Ang University Gwangmyeong Hospital, Gwangmyeong-si, Gyeonggi-do, Republic of Korea; b Division of Critical Care, Department of Surgery, Chung-Ang University Gwangmyeong Hospital, Gwangmyeong-si, Gyeonggi-do, Republic of Korea.

**Keywords:** critical care, heat stroke, hemophagocytic lymphohistiocytosis, septic shock

## Abstract

Hemophagocytic lymphohistiocytosis (HLH) is a syndrome with potentially fatal consequences that results from an excessive immune response caused by malfunctioning natural killer cells and cytotoxic T lymphocytes. Secondary HLH, which is the predominant type in adults, is associated with various medical conditions, including infections, malignancies, and autoimmune diseases. Secondary HLH associated with heat stroke has not been reported.

**Patient concerns:**

A 74-year-old male was admitted to the emergency department after being unconscious in a 42°C hot public bath. The patient was witnessed to be in the water for more than 4 hours. The patient’s condition was complicated by rhabdomyolysis and septic shock, which were managed with mechanical ventilation, vasoactive agents, and continuous renal replacement therapy. The patient also showed evidence of diffuse cerebral dysfunction.

**Diagnoses:**

While the patient’s condition initially improved, the patient developed a fever, anemia, thrombocytopenia, and an acute rise in total bilirubin, which, we suspected, was caused by HLH. Further investigations revealed elevated serum ferritin and soluble interleukin-2 receptor levels.

**Interventions:**

The patient received 2 cycles of serial therapeutic plasma exchange to lower the endotoxin burden. To manage HLH, high-dose glucocorticoid therapy was done.

**Outcomes:**

Despite the best efforts, the patient did not recover and expired from progressive hepatic failure.

**Lessons:**

We report a novel case of secondary HLH associated with heat stroke. Diagnosing secondary HLH can be difficult since clinical manifestations of the underlying disease and HLH may present simultaneously. Early diagnosis and prompt initiation of treatment is required to improve the prognosis of the disease.

## 1. Introduction

Hemophagocytic lymphohistiocytosis (HLH) is a potentially life-threatening syndrome characterized by an unopposed surge of immune response due to malfunctioning natural killer (NK) cells and cytotoxic T lymphocytes.^[1]^ HLH is classified as either primary or secondary. Primary HLH is associated with genetic abnormalities, and secondary HLH is associated with various medical conditions, including infections, malignancies, and autoimmune diseases.^[2,3]^ Diagnosing secondary HLH is often challenging because clinical manifestations of the underlying disease and HLH can present simultaneously. Unremitting fever, pancytopenia, hepatosplenomegaly, coagulopathy, and an elevation of proinflammatory cytokines are key clinical manifestations of HLH,^[4]^ while some patients may exhibit central nervous system involvement.^[5]^ Here, we present the case of a patient with HLH presumed to be associated with heat stroke. Heat stroke is a serious and potentially life-threatening condition, and clinical manifestations often include fever and altered mental status, thus making diagnosing HLH more difficult.^[6]^ HLH associated with heat stroke has not been reported before. Following the case report of the patient is a literature review that may provide insights into this rare condition. The written informed consent was obtained from the patient’s legal guardian.

## 2. Case report

A 74-year-old male was found unconscious in a 42℃ hot public bath. The patient had been in the hot water for more than 4 hours and was taken to the emergency department. The patient had no previous medical or familial history. The patient’s initial vital signs were as follows: blood pressure of 70/57 mm Hg, heart rate of 128 beats/minutes, respiratory rate of 32 times/minutes, a body temperature of 40.8°C, and oxygen saturation of 98%. Laboratory tests showed anemia (hemoglobin 10.9 g/dL), elevated aspartate transaminase (AST; 659 IU/L), elevated alanine transaminase (ALT; 509 IU/L), elevated creatinine (2.26 mg/dL), and normal white blood cell count (6.08 × 10^9^/L) with 8% segmented neutrophil. C-reactive protein was reported to be <1.0. Prothrombin time international normalized ratio and activated partial thromboplastin time were within their normal ranges (prothrombin time international normalized ratio 1.18, activated partial thromboplastin time 37.8 seconds). A computed tomography (CT) and diffusion magnetic resonance imaging of the brain were taken to exclude any cerebral parenchymal pathologies, and no specific parenchymal lesions were detected. The patient was diagnosed with heat stroke and was admitted to the intensive care unit (ICU) for further examination and management. The patient received active cooling with ice bags and intravenous fluid resuscitation initiated by emergency department.

During the first night in the ICU, despite successful cooling (esophageal probe temperature of 36.1°C) and continuous intravenous fluid resuscitation, the patient’s vital signs acutely deteriorated with severe shock (mean blood pressure lower than 65 mm Hg). A cardiac enzyme study revealed elevated high sensitivity troponin-I (658.55 pg/mL), creatine kinase-myoglobin binding (CK-MB; 6.4 ng/mL), and myoglobin (>1200 ng/mL). However, cardiogenic shock was ruled out by a bedside echocardiography. The patient required endotracheal intubation followed by support with mechanical ventilation due to respiratory failure. Empirical antibiotics (Ertapenem) were administered for probable septic shock with multiorgan failure after obtaining 2 sets of peripheral blood cultures. Epinephrine (1 mcg/kg/minutes), norepinephrine (0.5 mcg/kg/minutes), and vasopressin (0.03 IU/kg/hours) were administered intravenously to maintain mean blood pressure. Continuous renal replacement therapy was applied under the diagnosis of severe rhabdomyolysis accompanied by anuric acute kidney injury.

On the second day of hospitalization, laboratory results showed that the patient’s status was worsening. The laboratory findings during ICU admission are shown in Table 1. Growth of Bacteroides thetaiotaomicron, Prevotella copri, and Collinsella aerofaciens was reported in both pairs of peripheral blood cultures obtained before the start of empirical antibiotics. The initial history of the patient (hourly exposure to the hot public bath) made the possibility of blood culture contamination less likely, and the administration of ertapenem was continued. The patient did not show any brainstem reflex, and an electroencephalography (EEG) showed generalized slow theta to delta waves suggestive of diffuse cerebral dysfunction. Decreased free thyroxine was detected (0.46 ng/dL), and supplementation of glucocorticoid and levothyroxine was started. Since there was an ongoing need for inotropic and vasoactive agent support, 2 cycles of serial therapeutic plasma exchange were carried out to reduce the endotoxin burden and intravascular cellular constituents. After the plasma exchange, the requirement of inotropic and vasoactive support was dramatically decreased. AST, ALT, lactate dehydrogenase, high sensitivity troponin-I, and CK-MB began to fall after reaching a peak at HD 3 (AST 3233, ALT 2236, lactate dehydrogenase 2043, troponin-I 1860, CK-MB 47.1). However, myoglobin was sustained above the measurement limit (>1200 ng/mL), and total bilirubin started to rise above the normal range. Despite the recovery of the brainstem reflex, the patient was stuporous, and the EEG finding of diffuse cerebral dysfunction did not change.

**Table 1:** Laboratory findings of the patient during the ICU admission.

Laboratory results	HD 1	HD 2	HD 3	HD 4	HD 5	HD 10	HD 16	HD 26	HD 36	Reference values
White blood cells, 10^9^/L	6.08	7.66	5.44	11.18	9.84	26	10.47	7.87	20.17	3.5–9.5
Segmented neutrophils, %	8	56	78	87	96	89	93	87	95	40–70
Hemoglobin, g/dL	10.9	10.5	8.9	9.6	8.7	6.5	8.6	9.4	9.3	13–17
Platelet, 10^9^/L	142	93	44	23	50	45	72	53	109	140–400
CK, IU/L	989	1267	2756	2712	2856	1319				0–190
LDH, IU/L	911	3170	3524	2156	1945	961	593	555	710	0–225
CRP, mg/L	<1.0				21.7	81.7				0–5.0
AST, IU/L	659	3220	3233	3025	2338	169	91	112	162	0–40
ALT, IU/L	509	2430	2236	1660	1317	193	97	153	145	0–41
Gamma-GTP, IU/L	120	141	125	75	51	94	160	542	561	0–60
Total bilirubin, mg/dL	0.6	0.9	1.9	2.5	2.8	9.5	16.6	27.5	23.8	0–1.2
Direct bilirubin, mg/dL	0.2	0.5	1.4	1.8	1.7	8.7	15.7	25.1	22	0–0.3
Creatinine, mg/dL	2.26	2.43	2.75	2.27	2.21	1.78	1.56	0.99	0.72	0.70–1.20
Troponin-I, pg/mL	97.4	1141.8	1860.1	1374.6	1234.9	147.6				<34.2
CK-MB, ng/mL	2.7	12.5	47.1	38.6	17.2					<5.2
Myoglobin, ng/mL	>1200	>1200	>1200	>1200	>1200	>1200				<154.9
PT, INR	1.18	6.04	2.65	2.08	2.47	1.56	1.27	1.28	1.24	0.88–1.15
APTT, sec	37.8	> 180	90.3	77	70.6	54.4	46.5	57.1	57.2	29.1–44.7

ADM = admission, ALT = alanine aminotransferase, APTT = activated partial thromboplastin time, AST = aspartate aminotransferase, CK = creatinine kinase, CK-MB = creatinine kinase-myocardial band, CRP = c-reactive protein, FDP = fibrin degradation product, GTP = glutamyl transpeptidase, HD = hospital days, ICU = intensive care unit, IL = interleukin, INR = international normalized ratio, LDH = lactate dehydrogenase, PT = prothrombin time.

As the patient’s condition stabilized, inotropic and vasoactive supports were titrated. Unfortunately, the clinical course of the patient worsened. Total bilirubin elevated acutely, and the patient had a fever, anemia, and thrombocytopenia. HLH was suspected, and consultation with the department of haemato-oncology was requested on the fifth day of admission. Further investigation revealed decreased fibrinogen (75 mg/dL), elevated serum ferritin (18373.4 ng/mL), and elevated soluble interleukin (IL)-2 receptor (646 U/mL). Splenomegaly was not evident on the abdominal CT scan. Five findings met the 8 HLH-2004 criteria,^[7]^ and the patient was diagnosed with HLH. A bone marrow biopsy was not performed due to severe thrombocytopenia. The diagnosis of HLH was confirmed without bone marrow biopsy through a multidisciplinary consulting involving a hematologist. As the patient was suspected of secondary HLH, a wide range of etiologic causes were investigated. Human herpes viruses, including herpes simplex virus, cytomegalovirus, and Epstein-Barr virus, as well as viruses associated with local hemorrhagic fever syndromes, such as a severe fever with thrombocytopenia syndrome, hemorrhagic fever with renal syndrome, tsutsugamushi disease, and leptospirosis, were negative. Human immunodeficiency virus and tuberculosis were also negative.

Meanwhile, an abdominal CT scan was performed regarding the patient’s history. No sign of infection was present in the urinary tract, prostate, and lower gastrointestinal tract. Ascending colon diverticulitis without abscess or free perforation was found. However, expert consultation opposed the possibility that diverticulitis was the source of the sepsis. Inotropic and vasoactive agent support was discontinued on the eleventh day of hospitalization, and a tracheostomy was performed on the 16th day. Continuous renal replacement therapy was substituted with intermittent hemodialysis on the 36 day. Although the patient’s vital signs and laboratory results normalized, except for total bilirubin, the patient was unconscious. EEG reported continuous slow delta activity, implicating metabolic or toxic encephalopathy. Focal seizure activity was newly reported, and an antiepileptic agent was administered. Hepatic and central nervous system involvement of HLH was presumed, and the patient was transferred to a tertiary academic hospital for administering cytotoxic and immunosuppressive agents. The patient expired from progressive hepatic failure despite high-dose glucocorticoid therapy for secondary HLH on the 44th day of hospitalization.

## 3. Discussion

HLH is a potentially life-threatening syndrome characterized by an uncontrolled amplification of the immune response due to an innate or acquired defect of the granule-dependent cytotoxic function of NK cells and cytotoxic T lymphocytes. The inability to clear the antigenic stimulus results in unopposed proinflammatory cytokine release and macrophage activation leading to hemophagocytosis and multiorgan failure.^[1]^ HLH is classified as either primary (genetic) or secondary (acquired). Primary HLH is associated with gene mutations and is further classified into 5 subtypes of familial HLH, certain types of immunodeficiency syndrome, and Epstein-Barr virus-driven diseases, according to corresponding genetic abnormalities.^[8]^ Secondary HLH is most associated with infection (typically herpesviruses), malignancy, and autoimmune diseases.^[2,4]^ Recently, HLH has gained attention because the clinical manifestations are difficult to distinguish from severe sepsis, leading to underdiagnosis of the disease in ICU patients.^[9,10]^

The diagnosis of HLH is challenging. The criteria were revised in 2004, suggesting that an HLH diagnosis can be established if a molecular diagnosis is consistent with HLH or 5 of the 8 following diagnostic criteria are fulfilled: Fever; Splenomegaly; Bicytopenia (hemoglobin < 9 g/dL, platelet < 100 × 10^9^/L, neutrophil < 1.0 × 10^9^/L); Hypertriglyceridemia (≥ 3.0 mmol/L) or hypofibrinogenemia (≤ 1.5 g/L); Hemophagocytosis in the bone marrow, spleen, or lymph nodes; Low or absent NK cell activity; Hyperferritinemia (≥ 500 μg/L); Increased soluble IL-2 receptor levels (≥ 2400 IU/mL).^[7]^ However, these diagnostic criteria have several weaknesses. First, these criteria have been established using a pediatric population. Second, there is a high false-negative rate in the early stages of the disease process, and strict adherence to the criteria may result in a delay in treatment.^[11]^ Lastly, the assays for NK cell activity and soluble IL-2 receptor levels are not available in many hospitals. Another hurdle for diagnosing secondary HLH is that the clinical manifestations of the triggering disease and HLH can present simultaneously.

In our case, we diagnosed secondary HLH according to the HLH-2004 criteria. After the diagnosis of HLH, extensive microbiological cultures of blood, urine, and suspicious lesions were collected in an effort to identify the source of the infection. Furthermore, abdominal and neck CT scans with expert consultation were taken to find the infection source. However, we could not specify the source of the polymicrobial anaerobic infection, which is thought to be a trigger of secondary HLH. Our hypothesis is that secondary HLH was triggered by heat stroke induced endotoxemia, not by localized infection. In a patient with heat stroke, active thermoregulatory response with cutaneous vasodilation and compensatory splanchnic vasoconstriction occur to permit the heat to dissipated to the environment.^[12]^ The splanchnic hypoxia results in the generation of highly reactive oxygen and nitrogen species that accelerate mucosal injury, leading to hyperpermeability of the gut and intestine.^[13,14]^ Gram-negative bacteria may then leak into the portal circulation from the gut space. Observation of hyperpermeability of gut and endotoxemia are well established findings in patients with heat stroke.^[15,16]^ In brief, we believe that the growth of multiple anaerobic bacteria in the initial culture test in our patient, which is presumed to be a trigger for secondary HLH, is a consequence of heat stroke induced gut hyperpermeability. Our extensive microbiological and imaging workup results consolidate the hypothesis of heat stroke driven secondary HLH.

To our knowledge, secondary HLH induced by heat stroke has not been reported. One case reported an HLH diagnosis in a patient with severe burns as a result of exposure to scalding water.^[17]^ Authors concluded that secondary HLH was triggered by systemic inflammatory response to severe burns involving 38% of body surface area. However, our patient suffered from heat stroke and not severe burns because the temperature of the public bath was 42°C. On the other hand, there are possibility that the HLH preceded the visit to the hot public bath. The fact that the patient was in the hot bath for more than 4 hours suggests that the patient was already having trouble thinking clearly. According to a report of 30 HLH patients, 17 (56%) patients had central nervous system involvement, of which 14 already had neurological symptoms before the diagnosis, and 3 developed neurologic symptoms during the course of the disease.^[5]^ In several Asian cultures, including South Korea and Japan, people enjoy bathing in hot springs or public baths as a treatment for various ailments. Indeed, anemia and thrombocytopenia were observed from the first day of hospitalization. The body temperature was high on admission but was considered a consequence of the heat stroke. However, the patient did not have any known malignancy or autoimmune disease and family members denied the sign of infection prior to the visit to the hot public bath. It is not possible to determine which of heat stroke or HLH was acquired first without tissue confirmation at the time.

The goal of HLH treatment is to reverse the deleterious uncontrolled immune response. The 2004 treatment protocol includes high-dose corticosteroids, intravenous immunoglobulin, etoposide, and cyclosporin, followed by bone marrow transplantation.^[7]^ However, this protocol was only validated in pediatric patients, and the optimal treatment for secondary HLH in adults remains undetermined. In addition, the hepatotoxicity of cytotoxic agents in the context of acute liver failure, as presented in this case, further limits the treatment plan. Accompanying infections should always be considered because most agents for HLH treatment have immunosuppressive effects, which may lead to the acute exacerbation of sepsis. This case was also accompanied by sepsis caused by a polymicrobial anaerobic infection, which limited the treatment for HLH. Still, there are a few reports of biological agents for adults with refractory HLH, including rituximab, infliximab, and etanercept.^[2]^

Secondary HLH in adults has been reported to have an overall mortality rate of 41 to 75%.^[2,18]^ In a US cohort of 68 adults with HLH, the median overall survival was 4 months.^[19]^ Patients with malignancy had a worse prognosis compared to those without (median survival 2.8 months vs 10.7 months) whereas patients receiving an allogeneic stem cell transplant had most superior prognosis (median survival 21.5 months). In a European cohort of 162 adults with HLH, features associated with a poor prognosis in multivariate analysis were increasing age, decreasing platelet count, underlying lymphoma, and lack of inclusion of etoposide in the initial therapeutic regimen.^[20]^ The poor prognosis of adult HLH has been reported mainly due to the progression of multiple organ failure associated with delayed diagnosis and frequent complications, such as an infection during immunosuppressive or cytotoxic treatment.^[21]^ In our case, HLH was strongly suspected on the fifth day of hospitalization, and preemptive IV dexamethasone was started concurrently with a consultation with a haemato-oncologist. Since hepatic failure worsened despite the administration of the intravenous dexamethasone, the use of cytotoxic agents was considered, but treatment was limited due to the accompanying sepsis. In order to improve outcomes in HLH patients, prompt initiation of treatment following early diagnosis is required.^[22]^ Since there is little time to exclude the probability of other concurrent diseases when the patient’s condition deteriorates rapidly, risk-benefit considerations for immune-cytotoxic agents should be carefully weighed.^[23]^

## 4. Conclusion

Our clinical case is a novel report of secondary HLH triggered by a heat stroke. Since prompt initiation of treatment following early diagnosis can improve the prognosis of HLH, it is crucial to suspect HLH early when unexplained organ failure progresses.

## Author contributions

**Conceptualization:** Hyo Jin Kim, Kyoung Won Yoon.

**Data curation:** Wongook Wi.

**Formal analysis:** Wongook Wi.

**Investigation:** Wongook Wi.

**Methodology:** Hyo Jin Kim, Kyoung Won Yoon.

**Project administration:** Hyo Jin Kim.

**Resources:** Wongook Wi.

**Supervision:** Hyo Jin Kim.

**Software:** Wongook Wi.

**Validation:** Wongook Wi.

**Writing – original draft:** Wongook Wi.

**Writing – review & editing:** Hyo Jin Kim, Kyoung Won Yoon.
